# Sex-specific differences in the prognostic value of ischemic pre-hospital ECGs

**DOI:** 10.3389/fcvm.2026.1847639

**Published:** 2026-06-10

**Authors:** L. E. Sams, L. Bachinger, M. Maul, L. E. Villegas Sierra, M. Woerndl, J. Tauber, K. Mourouzis, F. Korovesis, M. Klemm, L. Freyer, S. Massberg, K. D. Rizas

**Affiliations:** 1 University Hospital Munich, Ludwig Maximilian University of Munich, Munich, Germany; 2 Munich Heart Alliance, German Centre for Cardiovascular Research (DZHK), Munich, Germany; 3 Klinikum Memmingen, Teaching Hospital of LMU Munich, Memmingen, Germany; 4 Department of Cardiology, Kantonsspital St. Gallen, St. Gallen, Switzerland

**Keywords:** acute coronary syndrome, ischemic ECG, morality, sex-differences, target lesion revascularization

## Abstract

**Background:**

The identification of myocardial infarction (MI) requiring target lesion revascularization (TLR) in women remains challenging.

**Objective:**

To investigate sex-specific differences in value of preclinical ischemic ECGs for identification of MI requiring TLR and mortality.

**Methods:**

Patients with suspected MI (ST-elevation MI [STEMI] excluded) and preclinical ECGs (1/2014–11/2021), who underwent coronary angiography (CA) were identified. Ischemic ECG was defined as composite of: ST-depression/elevation (not STEMI), T-wave inversion, left bundle branch block or ventricular fibrillation/tachycardia. The primary endpoint was MI requiring TLR, secondary endpoints were 30-day and 5-year all-cause mortality. The association between an ischemic ECG and endpoints was tested using logistic regression. Multivariable analysis was adjusted for age, previous percutaneous coronary intervention or MI, hypertension, diabetes, severely reduced left ventricular ejection fraction, renal failure and time to CA.

**Results:**

1,963 patients were included in the study (age 72 [IQR 61-80], 654 [33.3%] females). Men presented with an ischemic ECG (63 vs. 52%; *p* < 0.001) and need for TLR (68.4% vs. 53.9%; *p* < 0.001) more frequently than women. Ischemic ECG only predicted TLR in female patients (OR 1.86; 95% CI 1.38−2.54; *p* < 0.001 vs. OR 1.12; 95% CI 0.88–1.43; *p* = 0.351; *p*-interaction=0.012). This remained stable after adjustment using multivariable analysis (OR 1.79, 95%CI 1.22–2.60, *p* = 0.003). 30-day mortality was 139 (7%), 5-year mortality was 59%. There was a significant association between ischemic ECG changes and intrahospital and 5-year mortality in both sexes.

**Conclusion:**

An ischemic prehospital ECG predicts MI requiring TLR in female patients and identifies high-risk patients for mortality in both sexes.

## Introduction

Cardiovascular diseases such as myocardial infarction (MI) remain a leading cause of morbidity and mortality worldwide (1–4). The current guidelines for the management of acute coronary syndrome (ACS) recommend performing an ECG on every patient with suspected ACS in less than 10 min of first contact with medical professionals to promptly identify patients with ST-elevation myocardial infarction (STEMI) and indication for emergency-revascularization (1, 2). These first ECG-recordings hold a special place in diagnostics as they are usually obtained closely to symptom-onset and before or shortly after application of antithrombotic medication, such as heparin or aspirin (2).

However, two-thirds of patients presenting with ACS suffer from non-ST-elevation myocardial infarction (NSTEMI) and further management differs from STEMI patients (4). For patients with suspected NSTEMI, current guidelines recommend rule-in/rule-out algorithms based on multiple parameters such as repeated high-sensitivity troponin measurements, ECG-changes, and clinical presentation (2, 5). Although these algorithms have a high negative predictive value, recent literature reports, that over one third (36%) of rule-in patients are finally diagnosed with non-obstructive MI but nevertheless receive an invasive coronary angiography (CA) without the need of percutaneous coronary intervention (PCI) (6, 7).

Women often suffer from delayed diagnosis and treatment, as they more frequently present with atypical symptoms compared to men (2, 8, 9). Previous studies have reported delayed reperfusion therapy due to ECG misinterpretation in female patients with STEMI and data on reliability of ECG interpretation in female NSTEMI patients is lacking (10, 11). A recently published review on over 450 articles including patients with ACS confirmed that ACS management especially in early phases is mainly unfavorable to women (12). Diagnosis of type 1 MI is generally less common in females, but they suffer from increased mortality rate compared to men in case of type 1 MI (4, 13). In addition, women are prone to higher complication rates in the case of CA (8, 13–15). The goal of this study was to investigate sex-specific differences in the prognostic value of ischemic preclinical ECG to guide the correct identification of type 1 MI in patients undergoing CA because of suspected ACS.

## Methods

### Study population

**Figure 1:**
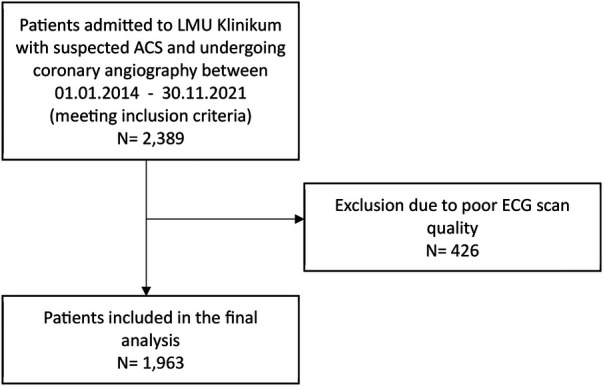
Inclusion and exclusion criteria; acute coronary syndrome (ACS), ST-elevation myocardial infarction (STEMI), coronary angiography (CA).

Between 1/2014 and 11/2021 we retrospectively identified patients with available pre-hospital ECGs, who underwent CA due to suspected ACS at two tertiary centers (LMU-university hospital Campus Großhadern and Campus Innenstadt) in Germany (Figure 1). In the case of repeated presentations, the first presentation was chosen. Prehospital ECGs were defined as ECGs performed by the emergency service before entering the hospital setting. We included patients ≥18 years of age (Figure 1). Patients with STEMI were excluded from the analysis (Figure 1). The study protocol was approved by the medical ethics committee of the Ludwig-Maximilians University, Munich, Germany (#21-1180).

### ECG analysis

Electronic scans of pre-hospital ECG recordings were obtained and screened for interpretability according to scan quality and length of the ECG recording. Manual ECG interpretation was performed by LB and LEVS. In case of uncertainty, ECG recordings were analyzed by two experts, KDR and LS, independently. A pathological (ischemic) ECG was defined as the composite of ST-depression, ST-elevation not meeting the criteria for STEMI or T-wave inversion in at least one ECG lead, left bundle branch block (LBBB) or ventricular fibrillation (VF)/ventricular tachycardia (VT). VT/VFs were only included in the analysis if STEMI was excluded at the first 12-lead ECG after cardioversion. LBBB was only included in this study, if the Sgarbossa criteria for STEMI were not met (16). SVTs without indication for coronary angiography were also excluded from this study. STEMI was defined using the current criteria suggested by the European society of cardiology guidelines for acute coronary syndrome, which use lower thresholds for female patients in leads V2 and V3 (for more details refer to Supplementary Material) (2).

### Assessment of baseline characteristics

LVEF was assessed using echocardiography with Simpon's method if available or visual assessment for ventricular function. LVEF was then stratified in to preserved (≥ 50%), mildly to moderately reduced (49%–30%) and severely reduced (≤30%). Angina pectoris (AP) pain was assessed on a scale of 0 to 4 following the Canadian Cardiovascular Society (CCS) Grading of Angina Pectoris (17).

### Endpoints

The primary endpoint of the study was a target-lesion revascularization (TLR), which was defined as immediate PCI or transfer to timely bypass-surgery. The secondary endpoints were 30-day intrahospital and 5-year all-cause mortality. Information about death was obtained using the official German death registry.

### Consent

The study protocol was approved by the medical ethics committee of the Ludwig-Maximilians University, Munich, Germany (#21-1180). Written informed consent was not obtained from the patients, as this is a retrospective analysis. Data were anonymized after completion of data acquisition.

### Statistical analysis

Continuous variables are presented as medians with Q1–Q3 quartiles and were compared using the Wilcoxon rank-sum test. Categorical variables are expressed as counts (percentages) and were analyzed using the chi-square test. Missing values were ignored. Differences in ECG parameters were assessed using logistic regression analysis, adjusted for age. The sex-specific association between subgroups and the primary and secondary endpoints were tested using logistic regression analysis and inclusion of the product of sex with the subgroup identifier. Subgroups included all components of the ischemic ECG, as well as age, aortic stenosis and left-ventricular ejection fraction (LVEF). Multivariable analysis was performed using Cox-regression analysis and was adjusted for age, previous PCI or MI, arterial hypertension, diabetes, severely LVEF, known renal failure maximum high sensitivity troponin before CA (max. 72 h before CA) and time to CA. The proportional hazards assumption was tested using diagnostics based on weighted residuals according to the method proposed by Grambsch et al. (cox.zph, survival package, version 3.4.0) (18). Landmark analysis was performed after 30 days. Survival analyses were performed using the Kaplan–Meier method. Sensitivity, specificity, positive and negative predictive values (PPV and NPV) were derived from the Kaplan-Maier curves with calculation of the corresponding confidence intervals by application of bootstrapping (*N* = 2,000). All analyses were performed using R Studio and CRAN R Version 4.5.2.

## Results

A total of 2,389 patients undergoing diagnostic CA due to suspected ACS were identified from 01.01.2014 to 30.11.2021 (Figure 1). Electronic scans of pre-hospital ECG data were available for these patients, but 426 patients had to be excluded due to poor ECG scan quality. Finally, 1,963 patients were included in the analysis.

The median age of the patients was 72 years (IQR 61–80), 654 (33.3%) of patients were females. Baseline characteristics varied significantly between female and male patients (Table 1). Female patients were older (76; IQR 67–82 vs. 70; IQR 59–79 years, *p* < 0.001; Table 1). Males presented with previous MI, coronary intervention, or previous bypass surgery more often than women (26.0 vs. 17.6%, *p* < 0.001; 38.0 vs. 26.0%, *p* < 0.001; and 7.1 vs. 2.1%, *p* < 0.001, respectively; Table 1). Male sex was associated with a significantly higher rate of cardiovascular risk factors such as hyperlipidemia (58.9 vs. 47.3%, *p* = 0.056) or smoking (51.2 vs. 28.8%, *p* < 0.001) and severely reduced LVEF <30% (11 vs. 6.6%, *p* = 0.002). In addition, CA was performed significantly later after admission in females than in male patients (median time to CA 14 3–42 h for females and 12 2–40 h for males, *p* = 0.042). On average, males were transferred to CA 2 h earlier than female patients (Table 2). High sensitivity troponin levels were similar in male and female patients and were significantly elevated in patients with ischemic ECG changes (Supplementary Table S7).

**Table 1:** Baseline characteristics.

Parameter	Male patients 1,309 (66.7)	Female patients 654 (33.3)	*p*-value
Age [median (IQR)]	70 (59–79)	76 (67–82)	<0.001
BMI [median (IQR)]	27 (25–30)	26 (23–30)	<0.001
AP pain [median (IQR)]	3 (2–3)	3 (2–3)	0.216
Preexisting conditions (*N*, %)			
Myocardial infarction	313 (26.0)	108 (17.6)	<0.001
Previous PCI	474 (38.0)	171 (26.0)	<0.001
Bypass	93 (7.1)	14 (2.1)	<0.001
Known kidney dysfunction	466 (37.3)	210 (33.4)	0.097
Cardiovascular risk factors (*N*, %)			
Positive family history	165 (16.8)	81 (15.9)	0.621
Hypertension	979 (90.0)	570 (89.1)	0.020
Hyperlipidemia	553 (58.9)	255 (47.3)	0.056
Diabetes mellitus	339 (31.0)	148 (26.2)	0.042
Nicotine	553 (51.2)	159 (28.8)	<0.001
Echo Parameters (*N*, %)			
LVEF ≥ 50%	775 (61.0)	419 (67.6)	0.006
LVEF 30–49%	355 (28.0)	160 (25.8)	0.325
LVEF < 30%	140 (11.0)	41 (6.6)	0.002
Aortic stenosis ≥ II°	71 (6.4)	33 (5.9)	0.682

BMI, Body-Mass-Indes; PCI, percutaneous coronary intervention, CAD, coronary artery disease; LVEF, left ventricular ejection fraction.

**Table 2:** Differences between male and female.

Parameter	Male patients	Female patients	*p*-value*
	1,309 (66.7)	654 (33.3)	
ECG changes (*N*, %)			
ST-depression	313 (23.9)	175 (26.8)	0.713
ST-elevation (non-STEMI)	400 (30.6)	170 (26.0)	0.099
T-wave inversion	460 (35.1)	208 (31.8)	0.003
SVT	175 (13.4)	104 (15.9)	0.938
LBBB	59 (4.5)	42 (6.6)	0.228
VT/VF	96 (7.3)	18 (2.8)	<0.001
Time to CA [median (IQR) in h]	12 (2–40)	14 (3–43)	0.042
CA performed	1,309 (100)	654 (100)	–
Ischemic ECG	**829** (**63.3)**	**341** (**52.1)**	**<0**.**001**
Target lesion requiring revascularization	**896** (**68.4)**	**353** (**53.9)**	**<0**.**001**
30-day intrahospital mortality	**99** (**7.6)**	**40** (**6.1)**	**0**.**068**
5-year mortality	**415** (**31.7)**	**213** (**32.6)**	**0**.**699**

*Logistic regression adjusted for age; CA, Coronary angiography; h, hours; STEMI, ST-elevation myocardial infarction; SVT, supraventricular tachycardia, LBBB, left bundle branch block; VT, ventricular tachycardia; VF, ventricular fibrillation.

An ischemic ECG was present in 1,170 (59.6%) patients. Males presented with an ischemic ECG more frequently than females (63.3 vs. 52.1%; *p* < 0.001), primarily driven by a higher rate of VT/VF and T-wave inversion (Table 2). In 1,249 (63.6%) cases a TLR was performed (Table 2). In 714 patients (36.4%) a type 1 MI requiring TLR could be ruled out after performance of CA. The final diagnoses of these patients are depicted in Supplementary Table S5 and included hypertensive heart disease (17.8%), atrial fibrillation or flutter (9.5%) or ischemic cardiomyopathy without the need of TLR (17.2%). Male patients more frequently presented with ischemic heart disease with intervention (20% vs. 12%) whereas female patients more commonly suffered from atrial fibrillation/flutter, Takotsubo cardiomyopathy or hypertensive heart disease (Supplementary Table S5).

A total of 139 patients died during the first 30 days of index hospitalization (male 7.6% vs. female 6.1%, *p* = 0.068; Table 2) and a total of 769 (58.7%) of patients died after 5-years of follow-up.

### Primary endpoint: association between ischemic ECG and TLR

In 1,249 patients (63.6%), a TLR was identified by CA (Table 2). TLR was more common in men than in women (68.4% vs. 53.9%; *p* < 0.001; Table 2).

**Figure 2:**
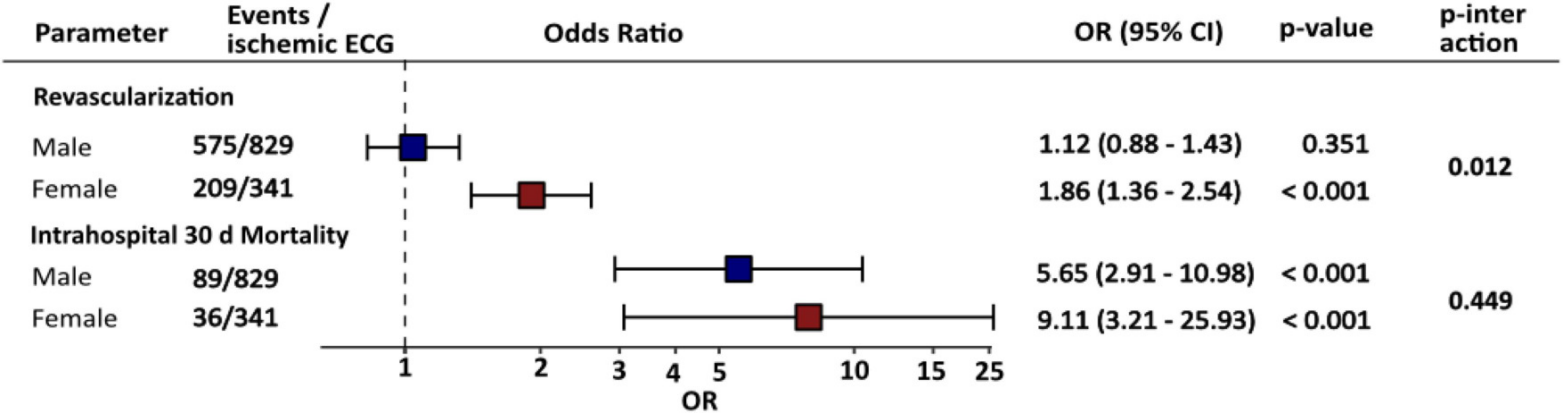
Primary and secondary endpoint, logistic regression analysis, odds ratio (OR), confidence interval (CI).

An ischemic ECG was associated with the presence of TLR in women (OR 1.86; 95% CI 1.36–2.54; *p* < 0.001), but not in men (OR 1.12; 95% CI 0.88–1.43; *p* = 0.351). The interaction between sex and the presence of an ischemic ECG was statistically significant (*p* = 0.012; Figure 2).

In females the rate of TLR was 61% (95% CI 56%–66%) if an ischemic ECG was present and 46% (95% CI 40%–52%) if not (*p* < 0.001; Supplementary Table S4). The association between ischemic ECG and TLR in females and males remained stable after adjustment for age, as well as other risk markers, including previous PCI, previous MI, arterial hypertension, diabetes, severely reduced LVEF, known renal failure, maximum high sensitivity troponin measured within 72 h before CA and time to CA (Supplementary Table S4).

### Secondary endpoint: association of ischemic ECG with intrahospital mortality

**Figure 3:**
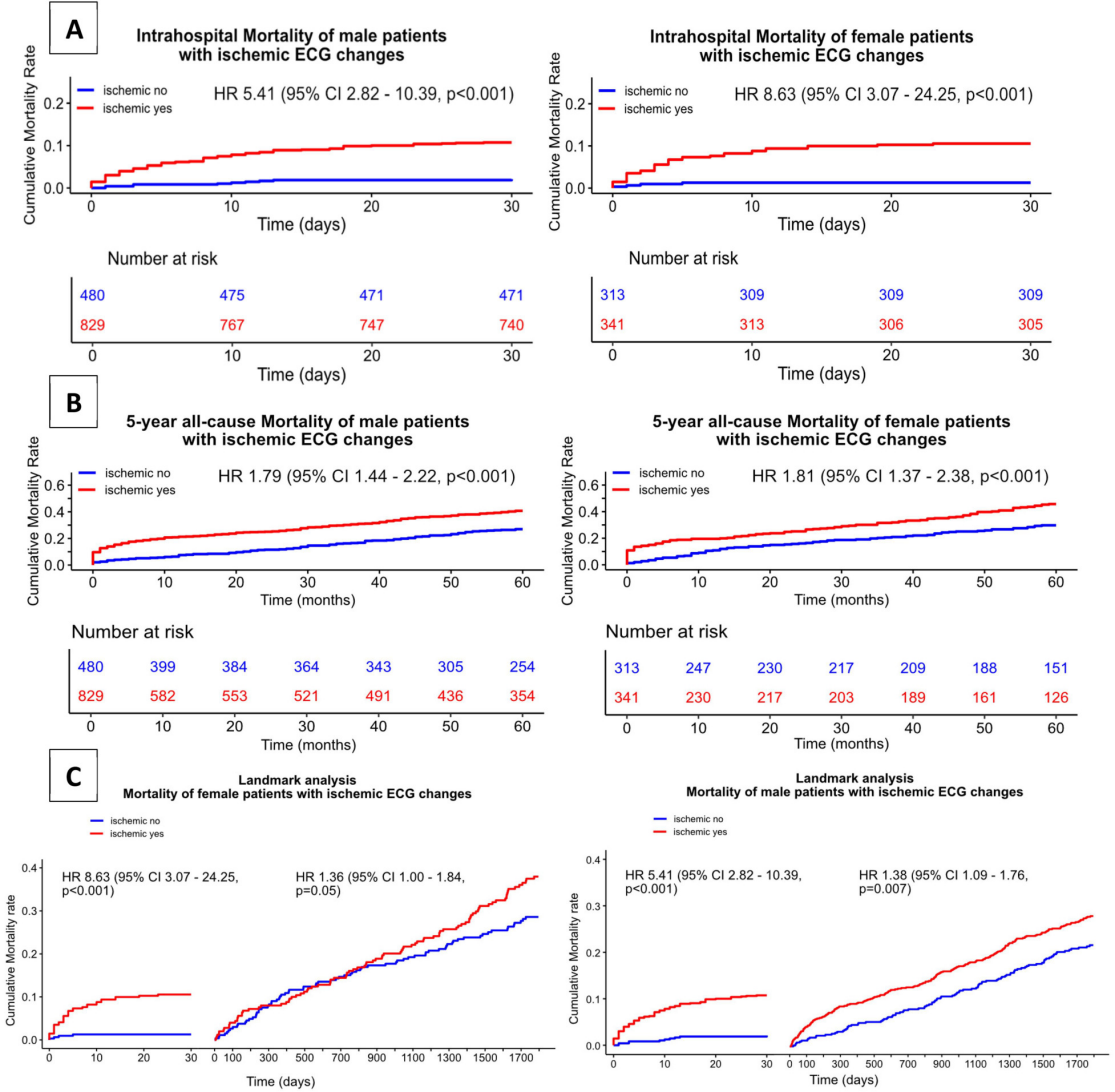
Kaplan meier curves depicting: **(A)**: 30-day intrahospital and **(B)**: 5-year mortality rates in male and female patients with and without ischemic ECG changes. **(C)**: Landmark analysis stratified before and after 30 days.

Compared to non-ischemic ECG, an ischemic ECG was significantly associated with increased mortality at 30 days (males OR 5.65; 95% CI 2.91–10.98, females OR 9.11; 95% CI 3.21–25.93; *p* < 0.001 for both; Figure 2). Kaplan Meier survival analysis showed a significantly increased mortality in patients with ischemic ECG changes (Figure 3). The proportional hazards assumption was met for female (*p* = 0.069), but not for male patients (*p* < 0.001). Therefore, landmark analysis was performed at 30 days, which showed that mortality risk depending on ischemic ECG changes was especially pronounced within the first 30 days and then significantly attenuated after that (HR for landmark analysis after 30 days: males HR 1.38;95%CI 1.09–1.76, *p* = 0.007, before 30 days: males HR 5.41;95%CI 2.82–10.39, *p* < 0.001; females after 30 days HR 1.36; 95%CI 1.00–1.84, *p* = 0.05, before 30 days: 8.63; 95%CI 3.07–24.25, *p* < 0.001). There was no significant interaction between the predictive value of ischemic ECG for mortality and sex (intrahospital mortality *p* = 0.449, 5-year mortality *p* = 0.959).

### Subgroup analyses

**Figure 4:**
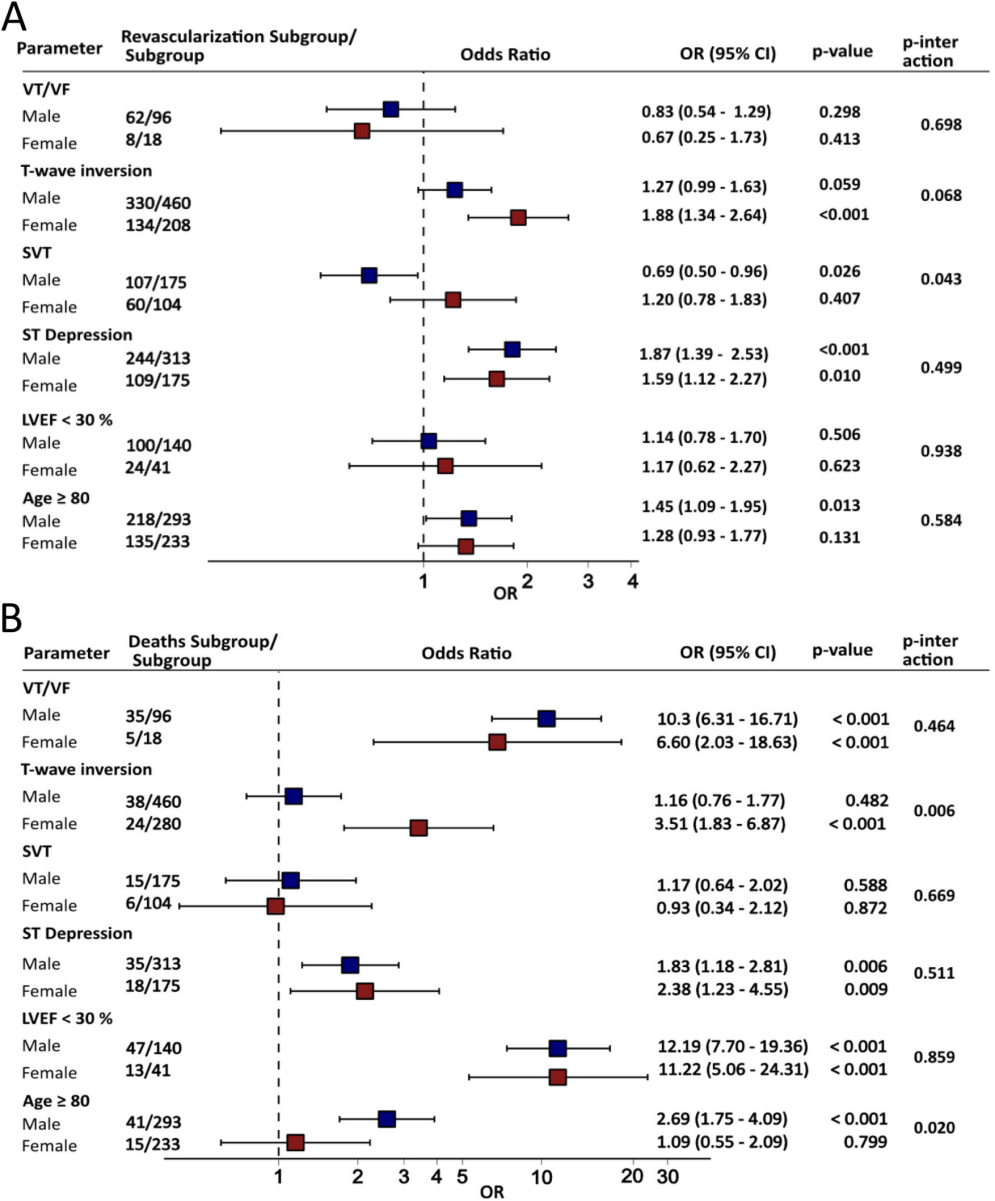
Subgroup analysis; **(A)**: odds ratio for revascularization depending on different subgroups; **(B)**: odds ratio for death depending on different subgroups. SVT, supraventricular tachycardia; LBBB, left bundle branch block, VT, ventricular tachycardia, VF, ventricular fibrillation, OR, Odds ratio, CI, confidence interval, LVEF, left ventricular ejection fraction.

The association between subgroups and the need for TLR, as well as 30-day intrahospital mortality for both sexes is illustrated in Figure 4 and Supplementary Tables S2, S3. T-wave inversion was predictive of the presence of TLR only in female but not in male patients (T-wave inversion: males OR 1.27; 95% CI 0.99–1.63, *p* = 0.059 vs. females OR 1.88; 95% CI 1.34–2.64; *p* < 0.001; *p*-interaction = 0.068, Figure 4;). This remained stable after adjustment for arterial hypertension in logistic regression analysis (OR after adjustment 1.85, 95% CI 1.31–2.60, *p* < 0.001) and interaction analysis showed no significant interaction between T-wave inversion and arterial hypertension (p_interaction_ = 0.995). In addition, female patients with ST-elevation not meeting STEMI criteria had an increased risk of TLR compared to male patients (Supplementary Table S2, OR females 1.54 95% CI 1.08–2.20, *p* = 0.018, *p*-interaction 0.009). There was also a significant interaction between females and males presenting with SVTs as females with SVTs had a significantly increased risk for the presence of TLR (OR females 1.20, 95% CI 0.78–1.83, *p* = 0.407, *p*-interaction=0.043). The presence of ST-depression was associated with an almost 2-fold increase of TLR in male and female patients (Figure 4). VT/VF and LBBB were not identified as significant predictors for the presence of TLR in male or female patients (Supplementary Table S2).

VT/VF, ST-depression and a severely reduced LVEF were highly predictive of mortality in male and female patients without significant interaction between the sexes (Figure 4B). T-wave inversion was highly predictive of intrahospital mortality in female but not in male patients (OR 3.51; 95% CI 1.83–6.87; *p* < 0.001 vs. OR 1.16; 95% CI 0.76–1.77, *p* = 0.482, *p*-interaction = 0.006; Figure 4B). Moreover, male patients presenting with moderate to severe aortic stenosis had an 8-fold increase of risk for intrahospital mortality (OR 7.64; 95% CI 3.79–15.42; *p* < 0.001; Supplementary Table S3). In our cohort, no females with moderate to severe aortic stenosis died during the index hospitalization (Supplementary Table S3).

## Discussion

This is the first study reporting the clinical importance of sex-specific differences of pathological prehospital ECGs in patients undergoing CA due to suspected ACS. Our results suggest that awareness regarding sex-specific differences in pre-hospital ECG changes can improve the identification of females in need of TLR. These findings are highly relevant, as females are severely underrepresented compared to disease prevalence in cardiovascular studies due to multiple social and biological factors (12, 19–21). Therefore, current guidelines are predominantly based on male population studies applied to the general population. This leads to underdiagnosis or a delay in diagnosis of MI in female patients, which is associated with increased morbidity and mortality (8, 9). In addition, our study confirms that females are transferred to CA later than male patients, which is in accordance with other studies (20).

In our cohort, the PPV of CA for predicting type 1 MI requiring TLR was comparable to other studies and reports (7). However, the rate of “unnecessary” CA, not leading to TLR was significantly higher in females than males (46.1 vs. 31.6%). Including the presence of an ischemic ECG significantly reduced the rate of unnecessary CA in women from 54 to 39%. This was independent from other risk factors such as age, previous PCI or MI, arterial hypertension, diabetes, severely reduced LVEF, known renal failure, maximum high sensitivity troponin measured within 72 h before CA and time to CA.

Among the various components of an ischemic ECG, T-wave inversion was highly predictive for type 1 MI requiring TLR in female patients. A recently published article on a small number of patients showed a significant prediction of TLR in patients with new onset T-wave inversion (22). As T-wave inversion is a common finding in hypertensive heart disease, the analysis was repeated with adjustment for arterial hypertension, after which T-wave inversion remained a significant predictor for TLR (23). ST-segment elevation not meeting STEMI criteria was predictive for type 1 MI in female patients in our cohort, although other studies have reported that non-specific ST-elevations can occur in females with non-obstructive myocardial infarction (MINOCA) or type 2 MI (24). Nevertheless, a recently published state-of-the-art review highlighted the importance of subtle ST-changes as 25% patients with acute coronary occlusion (ACO) do not present with a classical STEMI ECG (25). In accordance with previous studies, our results emphasize the importance of ST-segment depression as a predictor of relevant coronary occlusion in male and female patients (26). Our research adds a valuable contribution to the current understanding of ECG changes in patients with suspected ACS. Firstly, it emphasizes that sex-specific differences play an important role as ECG changes can improve the identification of type 1 MI in female patients significantly. In addition, due to the significant sample size, we were able to perform multivariable- and interaction analysis, which further enlightens the relevant topic of improving ACS diagnostics in female patients.

The presence of ischemic ECG changes was a highly significant predictor for intrahospital and 5-year mortality in female and male patients and was associated with an over 6-fold increase in intrahospital mortality. As reported in previous studies, we found that ST-depressions were associated with an increased mortality in female and male patients (26–28). In our cohort, T-wave inversion was a significant predictor for mortality only in female, but not male patients, which may explain why past studies showed inconclusive data regarding the prognostic value of T-wave inversions (27, 29). In our analysis, the presence of VT/VF was not predictive of TLR, which correlates well with previous large randomized trials, which did not show a benefit for early CA in patients with out-of-hospital cardiac arrest and missing signs of STEMI (30, 31).

As all patients in this analysis received CA and revascularization if needed, it remains unclear how to address this elevated mortality risk and further diagnostic and therapeutic methods should be explored to improve patient management. This is especially highlighted by the very divers final diagnoses found in patients without TLR which vary from ischemic heart disease without intervention to pulmonary embolism or hypertensive heart disease. Currently, performing CA remains an important step in the diagnostic algorithm of many of these diseases.

Nevertheless, our findings emphasize the importance of understanding differences in the presentation of female and male patients with suspected ACS and raise awareness when treating females with suspected ACS. Currently, there are great efforts to optimize the diagnosis and treatment of patients, who present with suspected ACS using ECG recordings (32). Especially the use of artificial intelligence has the potential to revolutionize identification of patients with suspected ACS (33–36). In addition, the use of ECG-based markers quantifying the tone of the autonomic nervous system could incrementally improve risk stratification strategies in patients following MI (37–40). Nevertheless, these methods are currently restricted to a limited number of specialized centers and are not reflected in guideline recommendations. Therefore, this analysis is of great importance for everyday clinical practice.

During the last 10 years several randomized controlled trials have tested the utility of CT-angiography to improve outcome and reduce unnecessary CA in patients with suspected ACS (41–43). These studies did not show a clinical benefit of early CT-angiography compared to standard care. However, none of these studies tested, whether the implementation of a two-step risk stratification algorithm with adoption of CT-angiography in patients with low pre-test probability, such as females with non-ischemic ECG, as well as the implementation of new techniques, such as calculation of CT-based fractional flow reserve can improve clinical outcome (44). A meta-analysis of existing randomized trials for subgroups meeting the above-mentioned criteria could probably enlighten this topic.

Our study has multiple limitations. Firstly, this study was performed at two centers associated with a single university hospital and therefore results may differ in other cohorts. Secondly, prehospital ECG-scans were only available for less than one quarter of the total cohort, which might have led to a selection-bias. Thirdly, non-traditional risk factors in females such as preeclampsia or early menopause were not collected but may play an important role in understanding ACS in female patients. In addition, no information about dynamic ECG changes during sequential ECG scans were available and finally, ECG interpretation was performed by visual analysis by two independent experts, but in general, results of ECG interpretation can differ from one to another specialist and therefore similar cohorts in other centers may present with different results.

In conclusion, the presence of ischemic changes, especially T-wave inversion, ST depression and non-significant ST-elevation on prehospital ECG predicts MI requiring TLR in female patients. Awareness regarding these findings may help to faster and more accurately identify female patients, who are in need of timely CA. In addition, ischemic ECG changes identify high-risk patients for mortality in both sexes.

## Clinical perspective

Ischemic ECG changes were associated with doubled rate of TLR in female, but not in male patients presenting with ACS and independent from known risk factors such as previous MI, hypertension or diabetes. Our results suggest that awareness regarding sex-specific differences in pre-hospital ECG changes can improve the identification of females in need of TLR and thus reduce the rate of unnecessary CA in women. Ischemic ECG changes were associated with an over 5-fold increase in intrahospital mortality in women and men. These patients should be considered for intensified monitoring.

## Data Availability

The raw data supporting the conclusions of this article will be made available by the authors, without undue reservation.
