# Supplementary material for: Sex-specific differences in the prognostic value of ischemic pre-hospital ECGs
Source: Front Cardiovasc Med. 2026 Jun 10;13:1847639. doi: 10.3389/fcvm.2026.1847639 (PMC13291123; doi:10.3389/fcvm.2026.1847639)
Supplement: Supplementary file 1 [file Datasheet1.pdf]

Supplementary Material

Sex-specific differences in the prognostic value of ischemic pre-hospital ECGs

Short title: Sex-specific predictive value of ischemic ECG

L E. Sams[1,2], L. Bachinger[1], M. Maul[1], L E. Villegas Sierra[1], M. Woerndl[1], J. Tauber[3], K. Mourouzis[1], F. Korovesis[1], M. Klemm[1,2], L. Freyer[1,2], S. Massberg[1,2], K D. Rizas[4,1,2]

(1) University Hospital Munich, Ludwig Maximilian University of Munich

(2) German Centre for Cardiovascular Research (DZHK), partner site: Munich Heart Alliance, Munich, Germany

(3) Klinikum Memmingen, teaching hospital of LMU Munich, Germany

(4) Department of Cardiology, Kantonsspital St. Gallen, St. Gallen, Switzerland

Table S1: primary and secondary endpoint, logistic regression adjusted for age

Group	Oddsratio	LCI	HCI	p-value	p-interaction
Revascularization male	1.10	0.87	1.41	0.423	0.018
Revascularization female	1.79	1.31	2.45	<0.001	
Death male	5.85	2.87	10.85	<0.001	0.469
Death female	8.83	3.10	25.12	<0.001	

Table S2: Complete Subgroup Analysis concerning TLR for patients with an ischemic ECG

Group	Events/ Subgroup	OR	LCI	HCI	p-value	p-interaction
**ST depression male**	244/313	1.87	1.39	2.53	<0.001	0.499
**ST depression female**	109/175	1.59	1.12	2.27	0.010	
**T wave inversion male**	330/460	1.27	0.99	1.63	0.059	0.068
**T wave inversion female**	134/208	1.88	1.34	2.64	<0.001	
**ST elevation male**	265/400	0.86	0.67	1.11	0.256	0.009
**ST elevation female**	105/170	1.54	1.08	2.20	0.018	
**LBBB male**	37/59	0.77	0.45	1.33	0.333	0.076
**LBBB female**	28/43	1.64	0.87	3.21	0.33	
**VT/VF male**	62/96	0.83	0.54	1.29	0.398	0.698
**VT/VF female**	8/18	0.67	0.25	1.73	0.413	
**SVT male**	107/175	0.69	0.50	0.96	0.026	0.043

**SVT female**	60/104	1.20	0.78	1.83	0.407	
**LVEF < 30 male**	100/140	1.14	0.78	1.70	0.506	0.938
**LVEF < 30 female**	24/41	1.17	0.62	2.27	0.623	
**AS ≥ Grade II male**	53/71	1.31	0.77	2.32	0.343	0.830
**AS ≥ Grade II female**	21/33	1.44	0.71	3.08	0.324	
**Age ≥ 60 male**	701/960	2.14	1.66	2.76	<0.001	0.975
**Age ≥ 60 female**	322/572	2.12	1.32	3.44	0.002	
**Age ≥ 80 male**	218/293	1.45	1.09	1.95	0.013	0.584
**Age ≥ 80 female**	135/233	1.28	0.93	1.77	0.131	

Table S3: Complete Subgroup Analysis concerning death for patients with an ischemic ECG

Group	Events/ Subgroup	OR	LCI	HCI	p-value	p-interaction
**ST depression male**	35/313	1.83	1.18	2.81	0.006	0.511
**ST depression female**	18/175	2.38	1.23	4.55	0.009	
**T wave inversion male**	38/460	1.16	0.76	1.77	0.482	0.006
**T wave inversion female**	24/280	3.51	1.83	6.87	<0.001	
**ST elevation male**	30/400	0.99	0.62	1.53	0.954	0.084
**ST elevation female**	16/170	1.99	1.01	3.82	0.040	
**LBBB male**	8/59	2.00	0.86	4.11	0.080	0.77
**LBBB female**	4/48	1.64	0.47	4.36	0.372	
**VT/VF male**	35/96	10.3	6.31	16.71	<0.001	0.464
**VT/VF female**	5/18	6.6	2.03	18.63	0.001	
**SVT male**	15/175	1.17	0.64	2.02	0.588	0.669
**SVT female**	6/104	0.93	0.34	2.12	0.872	
**LVEF < 30 male**	47/140	12.19	7.7	19.36	<0.001	0.859
**LVEF < 30 female**	13/41	11.22	5.06	24.31	<0.001	
**AS ≥ Grade II male**	13/71	8.14	3.9	16.28	<0.001	0.981
**AS ≥ Grade II female***	0/33	NA	NA	NA	NA	
**Age ≥ 60 male**	75/960	1.15	0.72	1.88	0.572	0.483
**Age ≥ 60 female**	37/572	1.82	0.64	7.67	0.327	
**Age ≥ 80 male**	41/293	2.69	1.75	4.09	<0.001	0.02
**Age ≥ 80 female**	15/233	1.09	0.55	2.09	0.799	

*no deaths occurred in this subgroup

Table S4 Positive predictive value for revascularization in patients with and without ischemic ECG

**WITHOUT ischemic ECG (95% CI)**		**WITH ischemic ECG (95% CI)**		**p-value**
Male	0.67 (0.63– 0.71)	Male	0.69 (0.66 – 0.73)	0.351
Female	**0.46** (0.40 – 0.52)	Female	**0.61** (0.56 – 0.66)	**<0.001**

Table S5 Primary diagnosis of patients without TLR

Diagnosis	N (%) of 714 patients	N (%) of male patients (413)	N (%) of female patients (301)
Peri-/Myocarditis	29 (4.0)	21 (5)	8 (3)
DCM	20 (3)	16 (4)	4 (1)
HCM	8 (1)	3 (<1)	5 (2)
Atrial fibrillation/flutter	68 (10)	33 (8)	35 (12)
Other SVT	24 (3)	13 (3)	11 (4)
VT or VF	64 (9)	47 (11)	17 (6)
Severe bradycardia	14 (2)	8 (2)	6 (2)
Stress-cardiomyopathy	16 (2)	1 (<1)	15 (5)
ICM without intervention	123 (17)	84 (20)	39 (12)
Hypertensive heart disease/crisis	127 (18)	54 (13)	73 (24)
Spontaneous Coronary Artery Dissection	3 (0.4)	2 (<1)	1 (<1)
Pulmonary embolism	8 (1.1)	4 (1)	4 (1)
Musculo-skeletal pain	19 (3)	16 (4)	3 (1)
Aortic stenosis	18 (3)	19 (5)	8 (3)
Infectious disease	32 (5)	20 (5)	12 (4)
Respiratory insufficiency	12 (2)	5 (1)	7 (2)
Neurological disease	16 (2)	8 (2)	8 (3)
Renal insufficiency	8 (1)	6 (1)	2 (1)
Other	89 (13)	55 (13)	34 (11)
No diagnosis	16 (2)	6 (1)	10 (3)

Table S6 Multivariable logistic regression analysis of TLR in female patients

**Parameter**	**Oddsratio**	**LCI**	**HCI**	**p-value**
Ischemic ECG	1.87	1.22	2.86	0.004*
Troponin T hs (cont.)	2.3	1.16	4.57	0.017*
Age (cont.)	1.01	0.99	1.03	0.481
Previous PCI	2.64	1.32	5.27	0.006*
Previous MI	1.17	0.51	2.65	0.709
Arterial hypertension	1.09	0.54	2.18	0.800
Diabetes	1.95	1.18	3.21	0.009*
Severely reduced LVEF	0.53	0.21	1.32	0.168
Known renal failure	1.49	0.93	2.39	0.097
Time to coronary angiography (cont.)	1	1	1	0.376

Table S7 Mean maximum Troponin values within 72 hours before coronary angiography

Subgroup	Mean maximum Troponin T hs in ng/ml
Males with ischemic ECG	0.472
Males without ischemic ECG	0.196
Females with ischemic ECG	0.452
Females without ischemic ECG	0.194

Definition of STEMI for male and female patients following the 2023 ESC guidelines for acute coronary syndromes

Contiguous Leads: New ST-segment elevation at the J point in 2 contiguous leads.
Leads V2-V3:

- ≥ 2.0 mm (0.2 mV) in men ≥ 40 years.
- ≥ 2.5 mm (0.25 mV) in men < 40 years.
- ≥ 1.5 mm (0.15 mV) in women.

Other Leads: ≥ 1.0 mm (0.1 mV) in all other leads (excluding V2-V3).
